# Smoking relapse reasons among current smokers with previous cessation experience in Shanghai: A cross-sectional study

**DOI:** 10.18332/tid/167963

**Published:** 2023-07-24

**Authors:** Ruiping Wang, Lingzi Shenfan, Yu Song, Qingliang Wang, Rui Zhang, Le Kuai, Bin Li

**Affiliations:** Clinical Research and Innovation Transformation Center, Shanghai Skin Diseases Hospital, Tongji University, Shanghai, China; School of Public Health, Shanghai University of Traditional Chinese Medicine, Shanghai, China; Department of Dermatology, Longhua Hospital, Shanghai University of Traditional Chinese Medicine, Shanghai, China; Department of Dermatology, Yueyang Hospital of Integrated Traditional Chinese and Western Medicine, Shanghai University of Traditional Chinese Medicine, Shanghai, China

**Keywords:** current smoker, relapse, smoking duration, smoking burden, smoking cessation

## Abstract

**INTRODUCTION:**

Quitting smoking can lead to substantial health gains, even later in life. Many smokers who attempt to quit experience several relapses before achieving sustainable cessation. This study aims to ascertain the differences between quitters with short and long abstinence time and to explore relapse reasons among smokers with cessation experience in Shanghai.

**METHODS:**

From January to December 2022, 1745 current smokers were recruited in Minhang, Jiading, Qingpu and Songjiang districts of Shanghai. We used an electronic questionnaire to collect data. We implemented logistic regression for odds ratio (OR) and 95% confidence interval (CI) calculation to explore factors associated with long cessation time among smokers with cessation experience of ≥3 months, ≥6 months, and ≥12 months.

**RESULTS:**

Of the 1745 smokers included, 1452 (83.2%) were males, with an average age of 44.2 years, and 48.0% (838/1745) had cessation experience but relapsed. Logistic regression indicated that smokers aged ≥45 years had a longer cessation duration (adjusted odds ratio, AOR=3.10; 95% CI: 1.97–4.88). Moreover, longer cessation duration among smokers was positively associated with low education level of junior high or lower (AOR=2.30; 95% CI: 1.42–3.72) and senior high (AOR=2.19; 95% CI: 1.53–3.15), older age at first tobacco smoking (AOR=1.62; 95% CI: 1.1.16–2.25), but was negatively associated with longer smoking duration (AOR=0.67; 95% CI: 0.43–0.00) and higher smoking burden (AOR=0.44; 95% CI: 0.28–0.72). The main reasons for cessation relapse were social interaction needs (34.5%) and discomfort due to abstinence (29.1%).

**CONCLUSIONS:**

The relapse rate was high among smokers even after 12 months of abstinence. Smokers with older age, lower education level, shorter smoking duration and lower tobacco burden had longer cessation duration. Social interaction needs and withdrawal symptoms were the main relapse reasons. It is highly recommended that health bureaux consistently conduct tobacco control initiatives to spread awareness about the detrimental effects of tobacco smoke and the advantages of quitting smoking, even after achieving cessation.

## INTRODUCTION

Smoking is the leading cause of preventable diseases, disability and death worldwide^1-3^. Recent data indicate that over 7 million people worldwide die each year due to smoking-related diseases^4,5^. In recent decades, tobacco smoking prevalence has declined gradually, but the World Health Organization (WHO) estimates that the population of current smokers is still over 1.1 billion worldwide^6^. So, decreasing tobacco smoking prevalence is still an important task and remains the crucial goal to save lives and reduce the disease burden for all countries globally^7^.

Tobacco control is an effective strategy to decrease the impact of smoking and can prevent the onset or progress of non-communicable diseases and reduce mortality^8^. Previous studies indicate that quitting smoking can lead to substantial health gains, even later in life^9^. Among smokers who quit, the incidence of cardiovascular diseases, cancers, and lung diseases and the overall mortality rate has decreased gradually since the cessation begins^10^. The death rate due to prolonged tobacco smoking can be reduced by up to 90% if the cessation is initiated before the age of 40 years among smokers^11^.

Despite the perceived benefits of smoking cessation, many smokers who attempt to quit smoking experience relapse^12^, and most smokers quit and relapse several times before they finally achieve sustainable cessation^13,14^. Previous studies indicate that smoking cessation relapse most frequently occurs within the first few weeks, and nearly 75% of smokers relapse within six months^12,15^. According to previous studies, individual, interpersonal, and organizational factors influence smoking relapse in quitters^16^. Moreover, the likelihood of relapse decreases with the elevated abstinence time, as approximately 70% of smokers quitting for over six months maintain smoking cessation for at least eight years^17^. In this view, a longer abstinence time predicts a lower relapse rate among quitters^15,17^. So, it is important to understand the differences between quitters with short abstinence time and those with long abstinence time before their relapses.

In this study, we implemented a cross-sectional study to learn about previous smoking cessation experience among current smokers, to understand the difference between quitters with short abstinence time and those with long abstinence time ahead of relapse, and to explore the reasons for relapse among current smokers with cessation experience in Shanghai, China.

## METHODS

### Study design

From January to December 2022, we conducted this cross-sectional study in Minhang, Songjiang, Qingpu and Jiading districts in Shanghai. In this study, the current smokers were a convenience sample selected based on the geographical representation but not a random sample of the entire smoking population in Shanghai. We applied a multistage sampling method to recruit smokers among the four districts as mentioned earlier in Shanghai. First, 8 out of 17, 5 out of 11, 7 out of 14, and 5 out of 10 sub-districts were selected randomly from Songjiang, Qingpu, Minhang and Jiading districts, respectively. Second, two residential blocks were randomly selected within each selected sub-district. Third, within each selected residential block, a complete list of home addresses of all households was compiled previously. A sample of 150 households was randomly extracted from the list without replacement. Fourth, the selected 150 households were ordered randomly, and each household was then approached sequentially until 35 current smokers were surveyed.

In light of the low smoking prevalence in females, one male and one female smoker from each selected household were surveyed, whenever possible. In this study, current smokers aged ≥18 years of both sexes were recruited, and smokers with neurological disorders, psychiatric abnormalities, or unable to provide informed consent were excluded. Finally, 1745 smokers in total completed the interview and were included in the data analysis.

### Data collection

In this study, we applied the ‘WenJuanXing’ electronic questionnaire to collect data. The online questionnaire has a quality control function that benefits data entry quality checkups and is convenient for paperless data input. The online questionnaire covered four parts: 1) demographic features including age, gender, marital status, education level, occupation, residency status and monthly income; 2) the history of non-communicable diseases (NCD) covering chronic bronchitis, chronic obstructive pulmonary disease, asthma, hypertension, type 2 diabetes, cancer, cerebroinfarction, and coronary heart diseases; 3) the tobacco smoking habits including age at first smoking, monthly tobacco expense, smoking cessation history, reasons for relapse, and tobacco smoking in the household and workplaces; and 4) the smoking cessation intention among smokers covering information on plans to quit smoking due to warning labels, retail cigarette prices, and physicians’ advice for quitting, respectively. A previous pilot study indicated that the split-half reliability coefficient of the questionnaire was 0.87, and the content validity coefficient was 0.85, which was reported in our previous work^18^.

### Index definition

In this study, a current smoker was defined as someone who smoked at least 100 cigarettes in a lifetime and still smokes just before the investigation^19-21^. Smoking cessation duration was defined as the interval between a quit attempt (from 1 day to several years) and relapse, where relapse essentially means commencing smoking after abstinence for a period^20-23^. In this study, smoking cessation duration was the combination of each cessation duration for smokers with more than one time of cessation experience, and then smoking relapses were evaluated at 3 months, 6 months and 12 months^24^. Smoking duration was defined as the time interval between investigation time and smoking initiation time among current smokers (removing the smoking cessation duration for those with cessation experiences)^20^ and was then classified as <20 or ≥20 years. We defined smoking intensity as the number of cigarettes smoked daily and categorized as <20 or ≥20 cigarettes/day. Smoking burden was calculated as the ratio of total monthly tobacco expense divided by monthly income, in percent, and categorized as <20% or ≥20%^21^. In this study, smokers’ age was classified as <45 years or ≥45 years, and we divided the marital status into unmarried and married. Education level was recorded as completed years of schooling and then classified as ‘junior high or lower’ (1–9 years of schooling), ‘senior high’ (10–12 years of schooling), or ‘college or higher’ (>12 years of schooling). We classified the occupation as laborers, professionals, or unemployed; individual monthly income was categorized as <5000, 5000–10000, or >10000 RMB^18^.

### Statistical analysis

The sample size in this study was calculated based on the formula for a cross-sectional study; we set the proportion of smokers with previous cessation experience among current smokers as 50%, α=0.05, δ=5% of p, and a non-response rate of 10%, requiring a total of 1690 current smokers to be recruited. In this study, SPSS 23.0 was employed for data analysis. Quantitative variables are presented as mean with standard deviation (SD) or median and interquartile range (IQR), as appropriate. We applied Student’s t-test or Mann–Whitney U tests to examine the differences between groups for quantitative variables. Qualitative variables were described as frequency and proportion (%), and chi-squared test was used for statistical significance tests between groups. Multivariable logistic regression was applied to calculate the adjusted odds ratio (AOR) and 95% confidence interval (95% CI) to explore factors associated with a longer smoking cessation duration among smokers with cessation experience categorized as ≥3 months, ≥6 months or ≥12 months. Confounders adjusted in the logistic regression model were those variables with p<0.05 identified through univariable analysis. Bar and box plots were produced to show the sex and age disparity for smoking cessation times and days of smoking cessation among smokers, respectively. A heatmap was used to show the difference in cessation relapse reasons among smokers by different demographic features and smoking habits. In this study, a difference with a p<0.05 (2-tailed) was regarded as statistically significant.

## RESULTS

Among 1745 current smokers in this study, the ages ranged from 18 to 75 years, with an average age of 44.2 years. Approximately 84% of smokers were males, and 86% were married. In this study, over 63% of smokers had an education level of college or higher, 45% were professionals, and only 25% had a monthly income <5000 RMB. Nearly 85% of smokers were local residents, and 22% had non-communicable diseases (NCDs). The proportions for smoking duration <20 years, smoking intensity <20 cigarettes/day, and smoking burden <20% were 41.7%, 60.3% and 76.6%, respectively (Table 1).

**Table 1:** The demographic characteristics among current smokers in Shanghai, China (N=1745)

*Characteristics*	*Total (N=1745) n (%)*	*Smokers with a previous quit attempt (N=838) n (%)*	*Smokers without a previous quit attempt (N=907) n (%)*	*χ^2^*	*p*
**Age** (years)				9.09	<0.001
<45	973 (55.8)	436 (52.0)	537 (59.2)		
≥45	772 (44.2)	402 (48.0)	370 (40.8)		
**Sex**				147.31	<0.001
Male	1452 (83.2)	792 (94.5)	660 (72.7)		
Female	293 (16.8)	46 (5.5)	247 (27.3)		
**Marital status**				22.03	<0.001
Unmarried	253 (14.5)	156 (18.6)	97 (10.7)		
Married	1492 (85.5)	682 (81.4)	810 (89.3)		
**Education level**				13.18	<0.001
Junior high or lower	213 (12.2)	95 (11.3)	118 (13.0)		
Senior high	424 (24.3)	236 (28.2)	188 (20.7)		
College or higher	1108 (63.5)	507 (60.5)	601 (66.3)		
**Occupation**				11.92	<0.001
Laborers	564 (32.3)	298 (35.6)	266 (29.3)		
Professionals	795 (45.6)	380 (45.4)	415 (45.8)		
Unemployed	386 (22.1)	160 (19.1)	226 (24.9)		
**Individual monthly income** (RMB)				7.13	0.028
<5000	442 (25.3)	189 (22.6)	253 (27.9)		
5000–10000	646 (37.0)	315 (37.6)	331 (36.5)		
>10000	657 (37.7)	334 (39.9)	323 (25.6)		
**Residency status**				13.89	<0.001
Local resident	1488 (85.3)	687 (82.0)	801 (88.3)		
Non-local resident	257 (14.7)	151 (18.0)	106 (11.7)		
**Non-communicable diseases** (NCD)				6.24	0.013
Yes	376 (21.55)	202 (24.1)	174 (19.2)		
No	1369 (78.45)	636 (75.9)	733 (80.8)		
**Smoking duration** (years)				0.18	0.669
<20	728 (41.7)	354 (42.2)	374 (41.2)		
≥20	1017 (58.3)	484 (57.8)	533 (58.8)		
**Smoking intensity** (cigarettes/day)				0.01	0.947
<20	1053 (60.3)	505 (60.3)	548 (60.4)		
≥20	692 (39.7)	333 (39.7)	359 (39.6)		
**Smoking burden** (%)*				68.92	<0.001
<20	1336 (76.6)	715 (85.3)	621 (68.5)		
≥20	409 (23.4)	123 (14.7)	286 (31.5)		

RS: respiratory system. NCD: non-communicable diseases. RMB: 1000 Chinese Renminbi about US$140.

* Smoking burden was calculated as the ratio of total monthly tobacco expense divided by monthly income, in percent.

### Characteristics of current smokers with cessation experience

In this study, 838 current smokers had previous cessation experience, the proportion was 48.1% (838/1745). Among the 838 smokers with cessation experience, the age ranged from 20 to 78 years, with an average of 43.7 years; 94.5% of them were males, and 81.4% of them were married. Over 60% of smokers with cessation experience had an education level of college or higher, and approximately 40% had a monthly income >10000 RMB. Table 1 shows that smokers with cessation experience were older, with a higher proportion of males and of unmarried status, with lower education level but higher monthly income, and had a higher prevalence of NCD than those without cessation experience. Moreover, smokers with cessation experience had slightly higher smoking intensity but lower smoking burden than those without cessation experience (Table 1).

**Figure 1:**
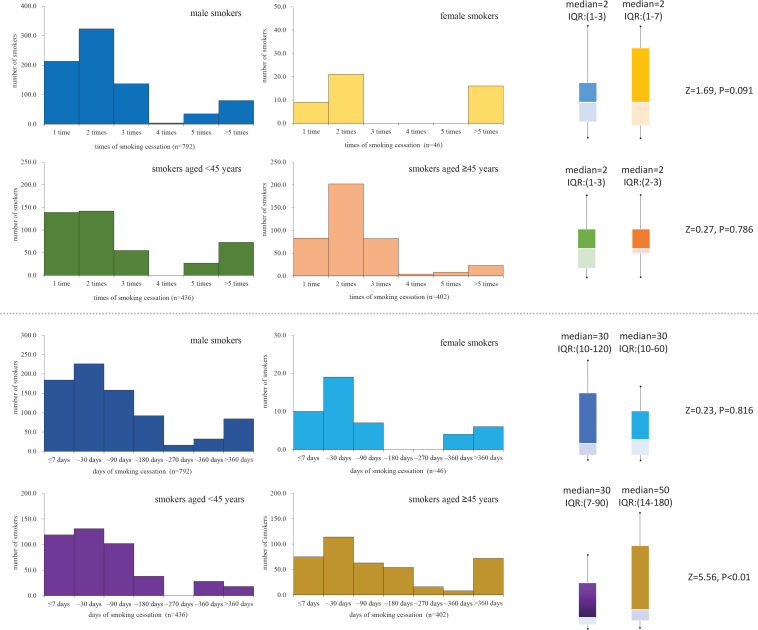
The sex and age disparity for tobacco smoking cessation times and days of smoking cessation among current smokers with previous cessation experience in Shanghai, China

Among the 838 smokers with cessation experience, the median value of cessation times was 2 (IQR: 1–3), and the smoking cessation days for the longest ranged from 1 to 2000, with a median value of 30 days (IQR: 10–120). Figure 1 shows that female smokers and smokers aged <45 years tended to have higher cessation times but without statistical significance. Moreover, male smokers had more smoking cessation days than female smokers but without statistical significance, whereas smokers aged ≥45 years had more than those aged <45 years (Figure 1).

### Tobacco smoking among current smokers with cessation experience

In this study, 838 smokers with cessation experience were divided into different groups: condition A (<3 and ≥3 months), condition B (<6 and ≥6 months), and condition C (<12 and ≥12 months), according to the days of smoking cessation. Compared with smokers with short smoking cessation duration (<3 months, <6 months, and <12 months), smokers with long smoking cessation duration had a lower percentage of age <45 years, had a lower proportion of an education level of college or higher, and had a higher prevalence of NCD. Smokers with long smoking cessation duration (≥3, ≥6 and ≥12 months) had delayed age at first tobacco smoking, with longer years of tobacco smoking and fewer daily consumed cigarettes, and had lower monthly expenses on tobacco purchase and lower proportion of smoking burden ≥20% than those with short cessation duration (Table 2).

**Table 2:** Demographic features, smoking initiation, smoking intensity, smoking duration among smokers with previous smoking cessation attempts in Shanghai, China

*Variable*	*Months of smoking cessation*					
	*<3 months (N=604) n (%)*	*≥3 months (N=234) n (%)*	*<6 months (N=696) n (%)*	*≥6 months (N=142) n (%)*	*<12 months (N=748) n (%)*	*≥12 months (N=90) n (%)*
**Age <45 years** ^† ǂ ‡ a^	352 (58.3)	84 (35.9)	390 (56.1)	46 (32.4)	418 (55.9)	18 (20.0)
Male smokers^a^	568 (94.0)	224 (95.7)	660 (94.8)	132 (92.9)	708 (94.7)	84 (93.3)
Married smokers^a^	484 (80.1)	198 (84.6)	564 (81.1)	118 (83.1)	607 (81.2)	75 (83.3)
**Education level** ^† ǂ ‡ a^						
Junior high or lower	48 (7.9)	47 (20.1)	76 (10.9)	19 (13.4)	80 (10.7)	15 (16.7)
Senior high	150 (24.8)	86 (36.8)	176 (25.3)	60 (42.3)	201 (26.9)	35 (38.9)
College or higher	406 (67.2)	101 (43.2)	444 (63.8)	63 (44.4)	467 (62.4)	40 (44.4)
**Smokers with NCD** ^ǂ ‡ a^	141 (23.3)	61 (26.1)	153 (21.9)	46 (34.5)	161 (21.5)	41 (45.6)
**Age of tobacco smoke initiation**^ǂ‡b^, median (IQR)	20 (18–25)	20 (18–25)	20 (18–25)	20 (18–28)	20 (18–25)	20 (18–23)
**Years of tobacco smoking**^†ǂ‡b^, median (IQR)	21.0 (11.5–28.0)	25.0 (19.0–35.0)	21.0 (12.0–28.0)	27.0 (19.0–34.0)	22.0 (13.0–28.0)	28.5 (19.0–35.0)
**Smoking duration** (years)^†ǂ‡a^						
<20	279 (46.2)	75 (32.1)	310 (44.5)	44 (31.0)	325 (43.5)	29 (32.2)
≥20	325 (53.8)	159 (67.9)	386 (55.5)	98 (69.0)	423 (56.5)	61 (67.8)
**Daily consumed cigarettes on average^b^,** median (IQR)	15 (8–20)	10 (8–20)	15 (8–20)	10 (5–20)	12 (8–20)	10 (8–25)
**Smoking intensity^a^** (cigarettes/day)						
<20	367 (60.8)	138 (58.9)	416 (59.8)	89 (62.7)	448 (59.9)	57 (63.3)
≥20	237 (39.2)	96 (41.1)	280 (40.2)	53 (37.3)	300 (40.1)	33 (36.7)
**Monthly expense on tobacco purchase^b^,** median (IQR)	500 (300–1000)	500 (300–1000)	500 (300–1000)	500 (300–1000)	500 (300–1000)	500 (300–800)
**Smoking burden**^ǂa^ (%)*						
<20	513 (84.9)	202 (86.3)	584 (83.9)	131 (92.3)	625 (83.6)	90 (100.0)
≥20	91 (15.1)	32 (13.7)	112 (16.1)	11 (7.7)	123 (16.4)	0 (0.0)

† The differences between smoker with previous smoking cessation <3 months and ≥3 months was statistically significant (p<0.05).

ǂ The differences between smoker with previous smoking cessation <6 months and ≥6 months was statistically significant (p<0.05).

‡ The differences between smoker with previous smoking cessation <12 months and ≥12 months was statistically significant (p<0.05).

a Chi-squared test between groups.

b Wilcoxon rank-sum test between groups.

* Smoking burden was calculated as the ratio of total monthly tobacco expense divided by monthly income, in percent. NCD: non-communicable diseases. IQR: interquartile range.

### Factors associated with longer cessation duration among smokers

In this study, logistic regression was used to explore factors associated with longer smoking cessation duration among smokers with cessation experience, for ≥3 months compared with <3 months (Model 1), ≥6 months compared with <6 months (Model 2), and ≥12 months compared with <12 months (Model 3) (Table 3).

**Table 3:** Factors associated with tobacco smoking cessation duration ≥3 months, ≥6 months or ≥12 months among smokers with previous smoking cessation attempts in Shanghai, China (N=838)

*Variable*	*Model 1*		*p*	*Model 2*		*p*	*Model 3*		*p*
	*AOR*	*95% CI*		*AOR*	*95% CI*		*AOR*	*95 % CI*	
**Age** (years)									
<45 (Ref.)	1.00	-		1.00	-		1.00	-	
≥45	**2.77**	**1.75–4.38**	<0.001	**4.79**	**2.63–4.49**	<0.001	**21.48**	**9.13–50.52**	<0.001
**Sex**									
Female	0.82	0.39–1.74	0.602	0.49	0.22–1.08	0.078	0.77	0.27–2.19	0.625
Male (Ref.)	1.00	-		1.00	-		1.00	-	
**Education level**									
Junior high or lower	**3.28**	**1.99–5.37**	<0.001	1.27	0.68–2.38	0.454	1.06	0.52–2.16	0.871
Senior high	**2.16**	**1.49–3.14**	<0.001	**2.28**	**1.47–3.53**	<0.001	1.45	0.84–2.49	0.183
College or higher (Ref.)	1.00	-		1.00	-		1.00	-	
**Smokers with NCD**									
Yes	-	-		1.44	0.94–2.22	0.095	**2.17**	**1.31–3.60**	0.003
No (Ref.)	-	-		1.00	-		1.00	-	
**Tobacco smoke initiation age** (years)									
<20 (Ref.)	1.00	-		1.00	-		1.00	-	
≥20	**1.59**	**1.14–2.22**	0.008	**2.17**	**1.45–3.23**	<0.001	**3.70**	**2.17–6.25**	<0.001
**Smoking duration** (years)									
<20 (Ref.)	1.00	-		1.00	-		1.00	-	
≥20	0.68	0.43–1.08	0.105	**0.51**	**0.28–0.92**	0.024	**0.15**	**0.07–0.32**	<0.001
**Smoking burden** (%)*									
<20 (Ref.)	1.00	-		1.00	-		-	-	
≥20	**0.56**	**0.35–0.91**	0.019	**0.21**	**0.11–0.43**	<0.001	-	-	

AOR: adjusted odds ratio. Model 1: Multi-variable logistic regression AOR of smokers with previous smoking cessation duration ≥3 months in comparison with smokes with smoking cessation duration <3 months; adjusted for age, gender, education level, age of tobacco smoking initiation, smoking duration, and smoking burden, that were selected through univariable analysis with a p<0.05. Model 2: Multi-variable logistic regression AOR for smokers with previous smoking cessation duration ≥6 months in comparison with smokes with smoking cessation duration <6 months; adjusted for age, gender, education level, NCD complication, age of tobacco smoking initiation, smoking duration and smoking burden, that were selected through univariable analysis with a p<0.05. Model 3: Multi-variable logistic regression AOR for percentage of smokers with previous smoking cessation duration ≥12 months in comparison with smokes with smoking cessation duration <12 months; adjusted for age, gender, education level, NCD complication, age of tobacco smoking initiation and smoking duration, that were selected through univariable analysis with a p<0.05. NCD: non-communicable diseases.

* Smoking burden was calculated as the ratio of total monthly tobacco expense divided by monthly income, in percent.

In Model 1, smokers aged ≥45 years had a higher proportion of smoking cessation duration ≥3 months (AOR=2.77; 95% CI: 1.75–4.38). In comparison with smokers who had an education level of college or higher, smokers with an education level of junior high or lower, or senior high, were more likely to have ≥3 months of smoking cessation (AOR=3.28; 95% CI: 1.99–5.37 and AOR=2.16; 95% CI: 1.49–3.14, respectively). Moreover, smokers with first tobacco smoking age ≥20 years were more likely to have smoking cessation duration ≥3 months (AOR=1.59; 95% CI: 1.14–2.22), and smokers with a smoking burden ≥20% were less likely to have smoking cessation duration ≥3 months (Table 3).

In Model 2 and Model 3, smokers aged ≥45 years had a higher proportion of smoking cessation duration of ≥6 months or ≥12 months (AOR=4.79; 95% CI: 2.63–4.49 and AOR=21.48; 95% CI: 9.13–50.52, respectively). Smokers with NCD had longer smoking cessation duration (AOR=1.44; 95% CI: 0.94–2.22, Model 2 and AOR=2.17; 95% CI: 1.31–3.60, Model 3). Moreover, smokers with first tobacco smoking age ≥20 years were more likely to have a longer smoking cessation duration, but smokers with a smoking duration ≥20 years and smoking burden ≥20% were less likely to have a longer smoking cessation duration (Table 3).

**Figure 2:**
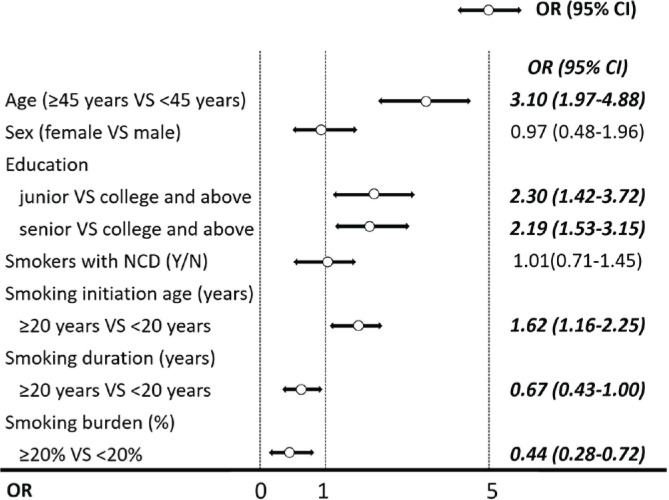
Forest chart for factors associated with longer tobacco smoking cessation duration (≥12 months, 6-11.9 months, 3-5.9 months, in comparison with <3 months) among smokers with previous smoking cessation attempts in Shanghai, China

To estimate the overall influencing factors for longer cessation duration among smokers, smoking cessation duration was categorized into four groups: <3 months (reference group), 3–5.9 months, 6–11.9 months, and ≥12 months. Ordinal logistic regression analysis indicated that smokers aged ≥45 years had longer cessation duration (AOR=3.10; 95% CI: 1.97–4.88). Moreover, longer cessation duration among smokers was positively associated with lower education level of junior high or lower (AOR=2.30; 95% CI: 1.42–3.72) and senior high (AOR=2.19; 95% CI: 1.53–3.15), older smoking initiation age (AOR=1.62; 95% CI: 1.1.16–2.25), but was negatively associated longer smoking duration (AOR=0.67; 95% CI: 0.43–0.00) and higher smoking burden (AOR=0.44; 95% CI: 0.28–0.72) (Figure 2).

### Reasons for smoking cessation relapse

Table 4 shows relapse reasons for the latest cessation attempt among smokers. The reasons for smoking cessation relapse among 838 smokers included social interaction needs (34.5%), discomfort due to abstinence (29.1%), mental or physical pressure (16.6%), body weight gain (3.9%) or other (15.9%). In this study, the main reason for smoking cessation relapse was social interaction needs and discomfort due to abstinence among smokers with different smoking cessation duration. The proportion of social interaction needs was higher among smokers with a smoking cessation duration ≥6 months, and the proportion of discomfort due to abstinence was higher among smokers with cessation duration <6 months, the difference being statistically significant.

**Table 4:** Main reasons for relapse among smokers with smoking cessation experience in Shanghai, China (N=838)

*Reasons of smoking cessation relapse*	*Total n (%)*	*Smokers with different months of smoking cessation*				*χ^2^*	*p*
		*<3 months (N=604) n (%)*	*3–5.9 months (N=92) n (%)*	*6–11.9 months (N=52) n (%)*	*≥12 months (N=90) n (%)*		
Social interaction needs	289 (34.5)	204 (33.8)	30 (32.6)	20 (38.5)	35 (38.9)	17.95	<0.001
Discomfort due to smoking abstinence	244 (29.1)	187 (30.9)	26 (28.3)	9 (17.3)	22 (24.4)		
Mental or physical pressure	139 (16.6)	98 (16.2)	13 (14.1)	14 (26.9)	14 (15.6)		
Others (curiosity, fashion, etc.)	133 (15.9)	99 (16.4)	19 (20.7)	5 (9.6)	10 (11.1)		
Gain of body weight	33 (3.9)	16 (2.6)	4 (4.4)	4 (7.7)	9 (10.0)		
Total	838 (100)	604 (100)	92 (100)	52 (100)	90 (100)		

**Figure 3:**
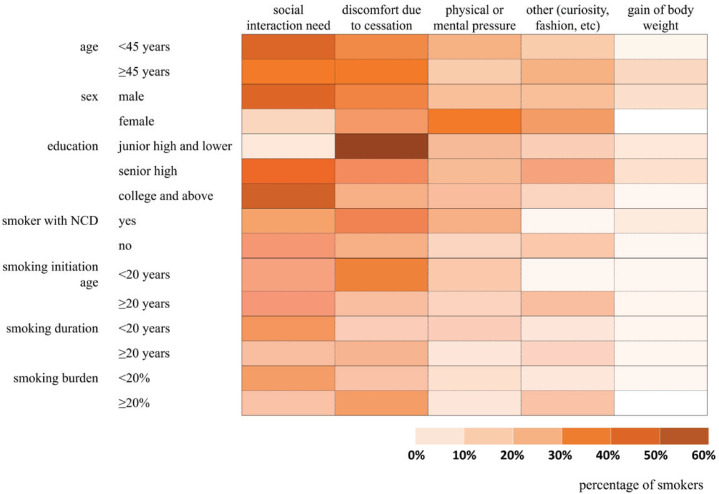
Heatmap for the proportion of relapse reasons among smokers with different demographic characteristics and smoking habits in Shanghai, China

In this study, the heatmap in Figure 3 indicates that the main reason for relapse among male smokers was social interaction need and cessation-induced discomfort, which was different in female smokers (mainly due to physical or mental pressure). Moreover, the main reason for relapse was the social interaction needs among smokers with an education level of senior high or higher but was cessationinduced discomfort among smokers with an education level of junior high or lower. There was no statistical difference in smoking relapse among smokers of different age, smoking initiation age, smoking duration, and smoking burden (Figure 3).

## DISCUSSION

Tobacco smoking is one of the most serious public health problems worldwide, and quitting smoking can lead to substantial health benefits^9,25^. Previous studies indicated that over 70% of smokers worldwide want to quit smoking, but most of them relapsed within six months, and even after two years of abstinence^23,26^. So, understanding the reasons for late relapse after a substantial period of quitting has been identified as an important public health issue^27^. In this study, we identified that the relapse rate within six months and 1 year were 83.1% and 89.3%, respectively. Smokers with older age (≥45 years), with lower education level (senior higher or lower), with NCD, with first tobacco smoking age ≥20 years, with smoking duration <20 years and smoking burden <20%, were likely to have a longer smoking cessation duration. The main reason for relapse among smokers with a smoking cessation duration of >6 months was social interaction need, which was different from the main relapse reason among smokers with a cessation duration of ≤6 months (discomfort due to abstinence).

Previous studies indicated that relapse rates within the first year of smoking cessation ranged from 60% to 90% worldwide^28^. In this study, the relapse rate of 3 months, 6 months, and 12 months was 72.1%, 83.1% and 89.3%, respectively, which was in line with previous studies^27^. Garvey et al.^29^ observed that nearly 90% of smokers who try to quit experience relapse within a year, and most relapse in the early weeks following the cessation effort^30^. Robinson et al.^31^ reported that over 50% of smokers with smoking cessation service relapse within the first year, and the relapse rate was even as high as 95% among non-treatment-seeking smokers^31,32^. Moreover, a substantial number of relapses occurred after the first year of abstinence^33^. Demographic features, high levels of nicotine dependence, more nicotine withdrawal symptoms, craving experiences, and low motivation to quit contributed to the smoking relapse among smokers^34,35^. So, we recommend that health bureaux promote smoking cessation and provide follow-up cessation services continuously among smokers who achieved cessation, even if the cessation duration was >1 year.

Age as a factor in smoking cessation is contradictory, some studies indicate the success of smoking cessation was among older adults with a lower relapse rate, but others found the opposite^36^. In this study, smokers aged ≥45 years had a higher proportion of smoking cessation attempts, and the proportions of smoking cessation duration for ≥3 months, ≥6 months and ≥12 months among smokers aged ≥45 years were higher than those aged <45 years, respectively. This might be due to the fact that older smokers were more concerned with their physical health, especially with the presence of chronic diseases. Previous studies reported that 60–70% of 1-year quitters maintained long-term abstinence of over five years^36^, so we can expect that the long-term relapse rate will be lower among smokers of older age. However, we expect that late relapse will still occur even after quitting for years among some smokers^22^. So, we should pay special attention to smokers aged <45 years who achieved cessation and provide continuous follow-up management for tobacco control among all smokers who recently achieved cessation despite their age.

In this study, we identified that smokers with an education level of senior high or lower were more likely to have longer smoking cessation duration than those with an education level of college or higher, in line with previous studies^16^. In Shanghai, smokers with lower education level were predicted to be older, and they were more concerned with their health. Moreover, female smokers who paid more attention to their health also tended to have an education level of senior high or lower. This could explain the longer smoking cessation duration among smokers with an education level of senior high or lower, to some degree. However, education level as a factor for smoking cessation success and long-term relapse needs further investigation.

Previous studies indicate that age at first smoking, nicotine dependence and personal economic status are associated with relapse among tobacco smokers with smoking cessation attempts^16^. In this study, smokers with age at first smoking <20 years and smokers with longer smoking duration (≥20 years) had short smoking cessation. This might be because smokers of younger age at first smoking and longer smoking duration tend to have high nicotine dependence. Nicotine in tobacco smoke is one of the most addictive substances; nicotine can enter the brain within seconds when exposed to tobacco smoke and act on the mesolimbic reward pathway; this causes dopamine release and produces a mood elevated physiologic response that becomes highly addictive. Meanwhile, the withdrawal symptoms can last for six weeks or longer, contributing to smoking cessation relapse. So, we should provide specific services (such as tobacco control clubs, smoking cessation campaigns, etc.) for smokers that have achieved cessation with a younger smoking initiation age and/or with longer smoking duration, and give essential medical assistance for those with heavy withdrawal symptoms to lower the relapse rate.

Withdraw symptoms, smoking habits of co-workers, craving experience, depression, anxiety, and other household smokers are associated with relapse^31,32^. In this study, smokers’ main reasons for cessation relapse were social interaction needs and withdrawal symptoms. Moreover, cessation relapse due to withdrawal symptoms was more common among smokers with cessation duration ≤6 months and the proportion of relapse due to social interaction needs was higher among smokers with cessation duration >6 months. Previous studies indicated that withdrawal symptoms were positively associated with relapse, and work and tobacco smoking were interconnected in many ways. Social interactions in the work environment might make it hard to quit and contribute to the high relapse rate, and the presence of smoking co-workers and the social atmosphere at the work unit also contribute to counteract cessation. So, we recommend that health bureaux implement health education and health promotion activities to advocate for a smoke-free household and smoke-free workplace, and an emphasis of the benefits of smoking cessation among smokers who attempt to quit would be also beneficial to prevent smoking relapse. Moreover, as a positive interaction tool, integrating tobacco control measures with social media (internet, TV, Twitter, newspaper, etc.) may prove even more effective in helping smokers to quit and preventing late relapse.

### Strengths and limitations

To our knowledge, this study is the first attempt to explore the differences in relapse reasons among smokers with short and long abstinence duration in Shanghai, China. This study has some limitations. First, the sample of current smokers was only selected in 4 suburb districts out of the total 15 districts in Shanghai, and current smokers selected by convenience sampling might limit the generalization of findings. Second, the smoking cessation relapse reasons were collected for the latest relapse because many smokers had more than one smoking cessation experience. So, the uncollected relapse reasons among smokers with repeated relapse experiences might induce some information bias. Third, the online electronic questionnaire for data collection was more convenient for data input but might have induced information bias to some degree because smokers, without the assistance of face-to-face interviews by trained collectors, might have had a different understanding of some of the questions. Fourth, except for the relapse reasons discussed in this study, multifaceted factors were associated with cessation relapse issues, including smoke-free households, clinician assistance, depression, decision latitude and reward. Fifth, in light of the low smoking prevalence in females in China, one male and one female smoker from each selected household were surveyed whenever possible. However, the underrepresentation of female smokers still hampers sex-specific conclusions. Sixth, this study used smoking intensity rather than FTND (Fagerström test for nicotine dependence) or HSI (heaviness of smoking index) to evaluate nicotine dependence, and the previous quit methods related to smoking cessation were not adopted. Therefore, the incorporation of these aforementioned factors would be a major step forward and should be considered in further studies.

## CONCLUSIONS

The relapse rate was high among smokers in Shanghai, even after 12 months of abstinence. Smokers of older age, lower education level, older age at first smoking, short smoking duration and lower tobacco burden had long cessation duration. Social interaction needs and withdrawal symptoms were the main relapse reasons for smokers with cessation duration >6 months and ≤6 months, respectively. We recommend that health bureaux continuously implement tobacco control activities to emphasize the harms of tobacco smoking and the benefits of cessation, and provide essential medical services among smokers even if cessation is achieved.

## Data Availability

The data supporting this research are available from the authors on reasonable request.
